# A Combined Study on the Use of the Child Behavior Checklist 1½–5 for Identifying Autism Spectrum Disorders at 18 Months

**DOI:** 10.1007/s10803-020-04838-0

**Published:** 2021-01-04

**Authors:** Natasha Chericoni, Giulia Balboni, Valeria Costanzo, Alice Mancini, Margherita Prosperi, Roberta Lasala, Raffaella Tancredi, Maria Luisa Scattoni, on behalf of the NIDA Network, Massimo Molteni, Giovanni Valeri, Stefano Vicari, Leonardo Zoccante, Maurizio Arduino, Paola Venuti, Carla Sogos, Andrea Guzzetta, Filippo Muratori, Fabio Apicella

**Affiliations:** 1 IRCCS Fondazione Stella Maris, Viale del Tirreno, 331, Calambrone, 56128 Pisa, Italy; 2 University of Perugia, Perugia, Italy; 3 Istituto Superiore di Sanità, Rome, Italy; 4 University of Pisa, Pisa, Italy

**Keywords:** Autism spectrum disorder screening, Baby sibling paradigm, CBCL 1½-5, Familial high-risk

## Abstract

The capacity of the Child Behavior Checklist 1½–5 (CBCL 1½–5) to identify children with autism spectrum disorder (ASD) at 18 months was tested on 37 children clinically referred for ASD and 46 children at elevated likelihood of developing ASD due to having an affected brother/sister. At 30 months the clinically referred children all received a confirmatory diagnosis, and 10 out of 46 siblings received a diagnosis of ASD. CBCL 1½-5 profiles were compared with a group of matched children with typical development (effect of cognitive level controlled for). The capacity of the CBCL 1½-5 DSM Oriented-Pervasive Developmental Problems scale to differentiate correctly between children diagnosed with ASD and children with typical development appeared dependent on group ascertainment methodology.

## Introduction

The Child Behavior Checklist (CBCL; Achenbach and Rescorla 2000) is a widely used parent-report checklist, which measures a broad range of behavioral and emotional problems. A number of studies have provided evidence of the utility of the CBCL in identifying children with autism spectrum disorders (ASD) at different ages (Biederman et al. 2010; Ooi et al. 2011; So et al. 2012). However, the majority of studies indicate that the CBCL 1½-5 might perform best in Level 1 screening, namely identifying potential cases of ASD in low risk populations, rather than in level 2 screening, among children referred for developmental evaluation. Indeed, the CBCL 1½-5 Pervasive Developmental Problems scale (PDP) and the Withdrawn Syndrome scale have shown a good sensitivity and specificity when children with ASD are compared with children with typical development (TD) (Havdahl et al. 2016; Limberg et al. 2017; Rescorla et al. 2015). However, specificity becomes suboptimal, meaning that there is a risk of over-identifying children with ASD (false positives) when the comparison group is composed of children with other behavioral, emotional, or developmental problems. For example, in Muratori et al. (2011), where the CBCL 1½-5 was used with three groups of children aged 24–60 months (101 diagnosed with ASD, 95 diagnosed with other psychiatric disorders (OPD), and 117 with TD), when the ASD group was compared with the TD group sensitivity/specificity values were 85%/90% for the DSM-PDP scale and 89%/92% for the Withdrawn scale. On the other hand, when the ASD group was compared with the OPD group, specificity was lower (60% for the DSM-PDP scale and 65% for the Withdrawn scale), indicating that some children in the OPD group had high scores on these scales even though they did not have ASD. It is noteworthy that sensitivity was unchanged (85% and 89%, respectively), indicating that both scales identified most of the children who received a diagnosis of ASD. So far, high sensitivity and specificity have been reported both in comparison with children with TD (n = 47) and children with OPD (n = 47) in only one study with young children with ASD (n = 47; age 18–36 months) (Narzisi et al. 2013). In this study, the comparison between the ASD group and the OPD group yielded a sensitivity of 0.85 and a specificity of 0.83 for the PDP scale and a sensitivity of 0.90 and a specificity of 0.83 for the Withdrawn scale. However, this optimal result was not replicated in the largest ASD screening study using the CBCL 1½-5 (Levy et al. 2019). In this study the DSM -PDP scale showed high sensitivity (80%) for identifying children with ASD (n = 656), whereas specificity varied depending on the comparison group (93% for 827 population controls, 85% for 646 children with developmental delay but no autistic features, and 50% for 284 children with developmental delay and autistic features). Thus, its utility as a level two screener needs to be further studied in order to understand with which clinical/at risk populations its specificity might be higher. Instead, its use as a level one screener has shown satisfactory levels for both sensitivity and specificity, suggesting its utility in routine developmental screening.

Since 2006 the American Academy of Pediatrics has recommended routine developmental screening with both broadband and autism-specific instruments at specified ages (Johnson and Myers 2007). Nevertheless, autism specific instruments are usually preferred. The most widely used autism specific screening tools are subsequent adaptations of the CHAT (Baron-Cohen et al. 2000), such as the Modified Checklist for Autism in Toddlers (M-CHAT; Robins et al. 2001). However, results on the sensitivity and specificity of these tools are not satisfactory. In one of the largest studies using the M-CHAT with a Follow-Up Interview (M-CHAT/F Robins et al. 2014) on a cohort of 25,999 children aged 16–26 months and followed-up through 4 to 8 years (Guthrie et al. 2019), the instrument yielded an overall sensitivity of 38.8%, and a positive predictive value (PPV) of 14.6%. When other developmental concerns were included as outcomes the PPV increased to 72.6%, however the sensitivity dropped to 11.7%, suggesting a limited utility of the M-CHAT/F for screening purposes.

Thus, for this purpose primary care practitioners might use broadband developmental screening tools rather than autism specific screening measures. If broad screeners were shown to be sensitive to autism, they could be used as a first level screen, while narrowband autism-specific screens could be used as a second level screen only for children with an autism risk indicated on the broadband screening (Hardy et al. 2015). In this regard, the broadband tool CBCL 1½-5 has shown high sensitivity and specificity as a first level screening tool, and the items of the PDP scale, revised with the publication of the DSM-5 and renamed ASD scale after removal of 1 item, are consistent with the DSM-5 diagnostic category of ASD (Achenbach 2014; Rescorla, Adams et al. 2019; Rescorla, Ghassabian et al. 2019a). Moreover, confirmatory factor analyses with data from population samples in 24 societies (*N* = 19,850) have shown good measurement invariance across societies (Rescorla, Adams et al. 2019). Compared to narrowband autism-specific screening tools the CBCL 1½-5 might offer several advantages as it requires minimal time commitment and cost. In addition it summarizes in a unique profile single behaviors pointed out by parents, identifying a wide range of behavioral and emotional problems, and it compares scores with normative data, limiting possible mistakes in the interpretation of results. Furthermore, as it contains a wide variety of behavioral/emotional problems, a parent’s pre-existing disposition to endorse or deny features of ASD may be less likely to influence ratings than might be the case on an ASD-specific instrument, and the age range covered spans the full period in which ASD is usually diagnosed, unlike many of the ASD-specific screening instruments (Rescorla, Winder-Patel et al. 2019b).

Few studies have tested the CBCL 1½-5 on clinically referred children with ASD as early as 18 months, mainly because families reach medical services when children are older (Ferrante et al. 2015; Garrido et al. 2018). However, improving early screening and diagnosis is fundamental because it means children can have an earlier access to intervention, which has been shown to significantly improve outcomes (Dawson et al. 2010; Wetherby et al. 2014). Consequently, establishing the efficacy of this instrument at a younger age would be of assistance to pediatricians in the early detection of children who need referral for diagnostic evaluation, as well as representing a valid support to clinicians in the diagnostic process.

In the past decade several studies with longitudinal designs have been implemented in order to study the development of ASD, identify specific precocious signs of the disorder and test early screening instruments (Zwaigenbaum et al. 2013, 2015; Costanzo et al. 2015). Many prospective studies have been conducted on children at familial risk for ASD due to an affected older sibling (Jones et al. 2014; Szatmari et al. 2016). Indeed, younger siblings of children with ASD are at a higher risk of developing ASD themselves: approximately 20% receive a diagnosis of ASD (Charman et al. 2016; Ozonoff et al. 2014). However, early diagnosis of ASD in children who may show sub-clinical ASD symptoms due to a familial genetic risk is quite complex. In their study of siblings at familial risk for ASD, Charman et al. (2016) found that among those who did not have an ASD outcome, around 11% had mild-to-moderate levels of developmental delay and 30% had high scores on the Autism Diagnostic Observation Schedule–2nd edition (ADOS-2; Lord et al. 2012). In these children who did not develop ASD, parents also reported high levels of ASD symptoms on the Autism Diagnostic Interview-Revised (ADI-R; Lord et al. 1994), as well as low adaptive functioning on the Vineland Adaptive Behavior Scales—2nd edition (Vineland-II; Sparrow et al. 2005). These findings on early emerging characteristics are an example of how complex an early diagnosis in infant siblings at familial risk for ASD can be.

As regards the use of the CBCL 1½–5 with younger siblings of children with ASD, Rescorla, Winder-Patel et al. (2019b) compared 56 2-year-old children at high risk for ASD with 26 low-risk children with an older sibling with TD. Consistently with previous studies, they found that the CBCL 1½–5 PDP scale and the Withdrawn syndrome scale differentiated well between children diagnosed with ASD and those not diagnosed. These data however were not replicated in another study performed by Nilsson Jobs et al. 2019, in which CBCL 1½–5 ratings by parents and preschool staff were compared in a sample of 46 3-year-old children at high risk for ASD and 14 low-risk TD controls. In their study, parent ratings were able to discriminate between groups that differed substantially in terms of symptoms (high-risk versus low-risk group), while they were less able to detect (or report) more subtle differences between affected and unaffected high-risk siblings. In contrast, preschool staff ratings were more accurate than parent ratings at differentiating children with and without ASD, and more closely associated with clinician-rated symptoms. In their discussion of the results, the authors hypothesized that parents’ reduced opportunity to observe different children’s behavior (compared to preschool staff) and the experience of an older child with ASD could bias parents’ ratings of the younger child.

Research on the CBCL 1½–5 as a tool to identify children with ASD among younger siblings of children with a diagnosis of ASD, is still quite limited. To this end, we evaluated the capacity of the CBCL 1½–5 to discriminate between children who were developing autism and their peers with typical development at 18 months of age.

In Study 1, we investigated the ability of the CBCL 1½–5 to discriminate children clinically referred for ASD at 18 months of age, who at 30 months received a confirmatory diagnosis of ASD, from children with TD matched for age and sex (cognitive level was controlled for). In Study 2, we investigated the ability of the CBCL 1½–5 to discriminate the following three groups: siblings of children with ASD at 18 months of age, who at 30 months received a diagnosis of ASD; siblings of children with ASD at 18 months of age, who at 30 months did not receive a diagnosis of ASD; and children with TD at 18 months. As in Study 1, the groups were matched for age, sex, and the effect of cognitive level was controlled for. In both studies further analyses were performed to assess correlation between parent ratings and clinicians’ observations, and ROC analyses were performed to evaluate the discriminative capacity of CBCL 1½-5-ASD related scales.

To our knowledge this is the first study to evaluate the capacity of the CBCL 1½-5 to discriminate children with ASD as early as 18 months. The inclusion of a group of children at familiar risk for ASD will contribute to the existing literature on sibling cohorts, where autism symptomatology can be expressed differently compared to clinically referred children who do not have familiarity for the disorder.

## Methods

### Participants

Three groups of children participated in this study (*n* = 142; all Caucasian). The first group was composed of 37 children with ASD (M/F = 32/5; age: *M [SD]* = 19.27 [1.41], range = 17–21 months) who were clinically referred to a tertiary-level hospital in Pisa (the Stella Maris Foundation) for an ASD evaluation (hereinafter CR-ASD) due to parental/clinical concern, prior to their second birthday. Parents filled in the CBCL 1½–5. Children underwent an extensive clinical assessment comprising a measure of autism symptoms (ADOS-2; Lord et al. 2012), a measure of the children’s level of development (Griffiths Mental Developmental Scales-Extended Revised; GMDS-ER; Griffiths 1996; Luiz et al. 2006), and adaptive functioning (Vineland-II; Sparrow et al. 2005) (see Table 1). Children were followed longitudinally until their third year of life, when a confirmatory diagnosis of ASD was obtained, based on gold standard tests, such as the ADOS-2 and the ADI-R, in accordance with the DSM-5 criteria (American Psychiatric Association, 2013). Only three children from our original sample (n. 40) did not receive a diagnosis of ASD (2 children had another neurodevelopmental disorder and 1 child did not receive any diagnosis) and were excluded from the analyses, as the number was not sufficient to create a comparison group of children with non-ASD diagnoses. The reason for this high rate of ASD diagnoses among clinically referred children is that the participants were selected at the Autism Spectrum Disorder Unit of an Italian Children’s Neuropsychiatric Hospital. All the children included in the study (n. 37) had idiopathic forms of ASD. Children with epilepsy, severe prematurity, known genetic syndromes such as Fragile X Syndrome, Rett and Down syndrome, did not take part.

**Table 1:** Mean (SD) of the scores obtained by clinically referred children for ASD (CR-ASD), siblings with a diagnosis of ASD (SIB-ASD), and siblings without a diagnosis of ASD (SIB-NonASD) on the ADOS-2, GMDS-ER, and Vineland-II scales

	CR-ASD (*n* = 37)		SIB-ASD (*n* = 10)		SIB-NonASD (*n* = 36)	
	*M*	(*SD*)	*M*	(*SD*)	*M*	(*SD*)
ADOS-2						
ADOS-2_Total	20.2	(5.40)	16.8	(4.37)	6.28	(3.93)
ADOS-2_CSS	7.9	(2.19)	6.7	(1.64)	2.75	(1.38)
GMDS -ER						
GMDS_General Quotient	79.0	(20.50)	78.2	(18.80)	102.0	(11.16)
Vineland-II						
Composite scale	80.0	(17.51)	84.4	(15.34)	99.3	(11.88)

The second group was composed of 46 children (M/F = 25/21; age: *M [SD]* = 18.37 [0.90]; range = 16–21 months) recruited in a sibling surveillance program (hereinafter SIB) as they had an affected older sibling. Parents filled in the CBCL 1½–5 and the children underwent a clinical assessment with the ADOS-2, the GMDS-ER and the Vineland-II at the Stella Maris Foundation. At the 30-month follow-up assessment SIB children were divided into two groups: 10 children (M/F = 7/3) obtained a diagnosis of ASD (SIB-ASD) while 36 children (M/F = 18/18) did not receive a diagnosis of ASD (SIB-NonASD) (see Table 1). The same exclusion criteria used for CR-ASD children were applied.

The control group consisted of 59 children with TD (M/F = 29/30), recruited specifically for this study in two kindergartens in a town in the center of Italy. The inclusion criteria for this group were: (i) age range 17–21 months; (ii) no parent or teacher concern about child development as noted in both of the two following descriptive items of the CBCL 1½-5: ‘Does the child have any illness or disability (either physical or mental)?’ and ‘What concerns you most about the child?’. For 27 of these children also a measure of cognitive development with the GMDS-ER was available (General Quotient *M [SD]* = 106.89 [9.55]).

To compare CBCL 1½-5 profiles between CR-ASD and children with TD, we selected the children in the TD group in order to match the 37 CR-ASD group for age and sex, following criteria proposed by Kover and Atwood (2013) for establishing equivalence in group-matching designs with participants with developmental disabilities (Cohen’s *d* [Cohen 1988] was evaluated as negligible, < 0.20, small, 0.20–0.50, medium, 0.50–0.80, or large, > 0.80). In this way, 37 children with TD were selected (M/F = 32/5; age: *M [SD]* = 19.30 [1.37]). The matched pairs did not differ in age (*Student’s t* = 1.00; *p* = 0.324, *d* = 0.16). Sex ratio was the same for both groups. For 27 pairs of children, it was also possible to compare the cognitive level, which resulted significantly lower in the clinical group compared to the group with TD (*Student’s t* = 6.15; *p* < 0.001; *d* = 1.41).

For the comparisons of study 2 (SIB-ASD vs. SIB-NonASD vs. TD), as the sibling groups with or without a diagnosis of ASD were of a different size and we could not use a matched-group design, we chose to include in the comparison analyses only the 27 TD children who had a cognitive measure (27 children with TD: M/F = 14/13; age: *M [SD]* = 18.37 [0.74]). A Chi-Square analysis was run to compare sex ratio across groups, and *W* was calculated to establish effect size (*W* was evaluated as negligible, < 0.10, small, 0.10–0.30, medium, 0.30–0.50, or large, > 0.50; Cohen 1988). Sex ratio was homogeneous across groups (*X*^*2*^ = 1.30; *p* = 0.52; *W* = 0.13). A Kruskall-Wallis test was used to compare age and cognitive level across groups, and *W* was calculated to establish effect size. Mean age did not differ significantly across groups (*X*^*2*^ = 2.24; *p* = 0.33; *W* = 0.17), whereas cognitive level differed significantly across groups (*X*^*2*^= 18.87; *p* < 0.001; *W* = 0.51). The Mann–Whitney test showed that the SIB-ASD group presented a lower General Quotient at the GMDS-ER both in comparison to children with TD (*Z* = 3.96; *p* < 0.001) and SIB-NonASD children (*Z* = 3.69; *p* < 0.001). Effect size, calculated using *r*_*g*_ was evaluated as negligible (< 0.10), small (0.10–0.30), medium (0.30–0.50), or large (> 0.50) following Cohen’s guidelines (1988) and was respectively 0.86 and 0.77. The two SIB groups differed significantly in their clinical profiles: statistical analysis performed with the Mann–Whitney test showed that the SIB-ASD group displayed more social-communicative impairments at the ADOS-2 (*Z* = 4.35; *p* < 0.001; *r*_*g*_ = 0.90) and lower adaptive functioning at the Vineland-II compared to the SIB-NonASD group (*Z* = 2.76; *p* = 0.006; *r*_*g*_ = 0.58).

### Measures

#### Child Behavior Checklist 1½-5 (CBCL 1½–5)

The CBCL 1½–5 (Achenbach and Rescorla 2000; Italian adaptation by Frigerio et al. 2006) is a standardized parent questionnaire, which examines a diversity of behavior and emotional problems in children from 1.5 to 5 years of age. It comprises 99 closed items, which describe a specific behavior and can be rated by parents on a three-point Likert scale based on the previous 2 months (0, not true; 1, somewhat or sometimes true; 2, very true or often true). There is also one open-ended item where any additional problems can be described by parents. The CBCL 1½-5 provides scores for three summary scales (i.e., Internalizing, Externalizing and Total Problems), five DSM-Oriented scales (i.e., Affective Problems, Anxiety Problems, Pervasive Developmental Problems, Attention Deficit/Hyperactive Problems and Oppositional Defiant Problems), and seven syndrome scales (i.e., Emotionally Reactive, Anxious/Depressed, Somatic Complaints, Withdrawn, Sleep Problems, Attention Problems, and Aggressive Behavior). *T* scores are available for each scale (*M* = 50; *SD* = 10). The CBCL 1½–5 has strong psychometric properties, including high test–retest reliability and Internal consistency (Achenbach and Rescorla 2000). After publication of the DSM-5, the DSM—Pervasive Developmental Problems scale was renamed DSM-Autism Spectrum Problems scale and one of the 13 items was removed (*n.3 Afraid to try new things*) (Rescorla, Adams et al. 2019; Rescorla, Ghassabian et al. 2019a). However, in our clinic we were still using the original 13-item DSM -PDP scale at the time of the study. As the two versions of the scale share 12 items, it is unlikely that results would have differed markedly (Rescorla, Winder-Patel et al. 2019b) (see in the Appendix Table 5 with the list of items of the DSM-PDP scale and their overlap with the Withdrawn Syndrome scale items).The CBCL 1½–5 has been translated into many languages and is used worldwide. In particular, the seven-syndrome model has been proved capable of describing preschoolers’ problems in very diverse societies, indicating possibilities for culture– general taxonomic constructs of preschool psychopathology (Ivanova et al. 2010).

#### Autism Diagnostic Observation Schedule–2nd Edition (ADOS-2)

The ADOS-2 (Lord et al. 2012; Italian adaptation by Colombi et al. 2013) is a semi-structured observation measure, used to assess communication, social interaction, and restricted and repetitive behaviors in individuals with ASD. For children under 30 months of age who have nonverbal mental ages of at least 12 months, the ADOS-2 Toddler Module is used. It provides three ranges, which are associated with the need for clinical monitoring and follow-up, and indicate little-or-no, mild-to-moderate, or moderate-to-severe concern (Luyster et al. 2009). It is also possible to calculate a calibrated severity score (CSS) for the total score (range: 0–10), which provides further information on the severity of the disorder.

#### Griffiths Mental Developmental Scales-Extended Revised (GMDS-ER)

The GMDS-ER (Griffiths 1996; Luiz et al. 2006) assesses child development through 6 sub-scales: Locomotor, Personal-Social, Language (receptive and expressive vocabulary), Eye and Hand Co-ordination, Performance (Visuospatial skills including speed of working and precision) and Practical Reasoning (the latter is used only with children above two years of age). A general quotient (*M* = 100; *SD* = 15) can be calculated combining the scores from the subscales. The GMDS-ER has been administered across a variety of clinical populations and has proved to be an effective and efficient tool in a diversity of cultural and social contexts Jacklin and Cockcroft 2013).

#### Vineland Adaptive Behavior Scales—2nd Edition (Vineland-II)

The Vineland-II (Sparrow et al. 2005; Italian adaptation by Balboni, Belacchi et al. 2016a, b) is a semi-structured parent interview designed to assess adaptive functioning across four subdomains—communication, daily living, socialization, and motor skills. Standard scores (*M* = 100; *SD* = 15) can be obtained for each domain and combined to provide an adaptive behavior composite standard score. The scale has optimal reliability and concurrent validity, and has been widely used in clinical and research settings with children on the autism spectrum, proving to be sensitive to the specific impairments experienced by these children (Balboni, Tasso et al. 2016a, b; Perry et al. 2009; Ray-Subramanian et al. 2011).

### Procedures

The study was carried out from 2016 to 2019 in accordance with the standards for good ethical practice of the Stella Maris Foundation. Written informed consent was obtained from all the children’s parents. Parents in the TD group filled in the CBCL 1½-5 anonymously at kindergarten. Parents of the CR-ASD group filled in the CBCL 1½-5 during an extensive clinical assessment. Parents of SIB children filled in the CBCL 1½-5 within a surveillance research program, which comprised a number of screening and diagnostic measures.

### Data Analysis

In study 1 differences in CBCL 1½-5 scales across CR and TD groups were investigated using Student’s Paired Samples t-test. The level of significance was set at *p* < 0.003 in accordance with Bonferroni’s correction for multiple comparisons (0.05/15 = 0.003) and Cohen’s *d* (1988) was calculated to evaluate the effect size of significant differences. Regression analyses were performed to examine the capacity of the CBCL 1½-5 Withdrawn and PDP *T* scores to predict diagnostic status as well as to verify the role played by cognitive level. Diagnostic status was set as a dependent variable while the Withdrawn and PDP scale scores were entered separately in the regression analysis and set as predictors, together with the cognitive level. The significance of the models as well as the percentage of correctly identified children were computed and *β* coefficients were calculated to verify the contribution of each independent variable to the model.

The association between parent CBCL 1½-5 ratings and the ADOS-2 clinical assessment at 18 months (ADOS-2 total score) was assessed using Pearson’s correlation. Finally, because the Withdrawn and PDP scales have been identified in the literature as the best predictors of the presence of ASD, we used ROC analyses to estimate the diagnostic accuracy of these scales in our sample of clinically referred children for ASD.

In study 2, as assumptions for parametric tests were not met, differences in CBCL 1½-5 scales were investigated using the Kruskal–Wallis test (SIB-ASD vs. SIB-NonASD vs. TD). To control for the effect of cognitive level, standardized residuals of each CBCL 1½-5 scale *T* score were computed with cognitive level as the independent variable and each CBCL 1½-5 scale *T* score as the dependent variable. These scores were then used to compare CBCL 1½-5 scales across groups. The level of significance was set at *p* < 0.003 in accordance with Bonferroni’s correction for multiple comparisons (0.05/15 = 0.003).

The association between parent CBCL 1½-5 ratings and the ADOS-2 clinical assessment at 18 months (ADOS-2 total score) was assessed using Spearman’s correlation.

## Results

### Study 1: CR-ASD vs. TD

#### Comparison Between CBCL 1½-5 Profiles

**Fig. 1:**
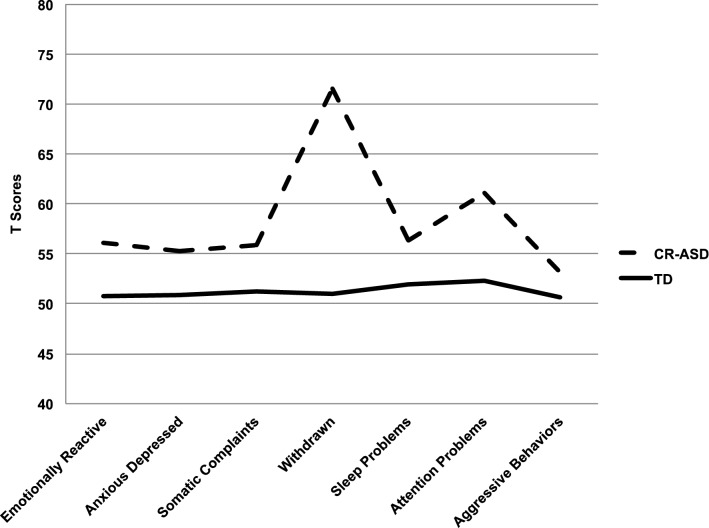
CBCL 1½-5 profiles across syndrome scales in clinically referred children with ASD (CR-ASD) and children with typical development (TD)

CR-ASD children scored significantly higher than the TD group on all scales except for the Sleep Problems and Aggressive Behaviors scales (*p* ≤ 0.003; see Table 2). Furthermore, in the CR group the Withdrawn scale and the PDP scale obtained higher scores compared to the other scales, with scores above the clinical cut-off of 70 for the Withdrawn scale (see Fig. 1) and in the borderline range (> 65) for the PDP scale. The effect size for both scales was large (*d* > 1).

**Table 2:** Comparison across clinically referred children for ASD (CR-ASD) and children with typical development (TD) on the T scores of the CBCL 1½-5 scales: means, standard deviations, Student’s t value and Cohen’s d effect size

	CR-ASD		TD				
	(*n* = 37)		(*n* = 37)				
	*M*	*(SD)*	*M*	*(SD)*	*Student’s t*	*p value*	*Cohen's d*
Syndrome scales							
Emotionally reactive	56.08	(6.95)	50.78	(2.88)	4.20	< 0.001*	0.69
Anxious depressed	55.27	(6.33)	50.92	(2.34)	3.72	0.001*	0.61
Somatic complaints	55.84	(6.77)	51.24	(3.53)	3.40	0.002*	0.56
Withdrawn	**71.51**	(14.59)	51.03	(2.58)	8.67	< 0.001*	1.43
Sleep problems	56.3	(9.10)	51.95	(3.28)	2.62	0.013	0.43
Attention problems	61.11	(8.44)	52.32	(3.54)	5.70	< 0.001*	0.94
Aggressive behaviors	53.16	(4.75)	50.62	(1.32)	3.12	0.004	0.51
Broadband scales							
Internalizing problems	58.54	(11.30)	41.14	(7.69)	8.03	< 0.001*	1.32
Externalizing problems	51.95	(8.77)	43.62	(6.91)	4.65	< 0.001*	0.76
Total problems	56.11	(11.38)	41.86	(6.75)	6.93	< 0.001*	1.14
DSM-oriented scales							
DSM-Affective	59.05	(8.58)	51.41	(2.71)	5.11	< 0.001*	0.84
DSM-Anxiety	55.27	(7.69)	50.73	(2.28)	3.34	0.002*	0.55
DSM-PDP	**67.49**	(11.73)	51.46	(3.32)	8.12	< 0.001*	1.34
DSM-ADH	56.68	(6.63)	52.19	(3.44)	3.60	0.001*	0.59
DSM-ODP	53.3	(4.81)	50.46	(0.99)	3.48	0.001*	0.57

Scores above the clinical/borderline cutoff are evidenced in bold. The level of significance was corrected for multiple comparisons in agreement with Bonferroni’s procedure (*p < 0.003)

#### Regression Analyses

Logistic regression analyses performed to assess prediction of diagnostic status (ASD vs. NonASD) based on scores from the Withdrawn CBCL 1½-5 scale and the GMDS-ER general quotient set as predictors showed a good model fit (*X*^2^ = 37.47, *p* < 0.001; Nagelkerke *R*^2^ = 0.69). The model was able to classify correctly 86.8% of the children. However, only the Withdrawn scale proved to be a critical predictor of diagnostic status with a *β* coefficient of 0.24 (*p* = 0.02), whereas the contribution of the cognitive level was not significant, with a *β* coefficient of − 0.44 (*p* = 0.15).

Also the results of the regression analysis with the PDP CBCL 1½-5 scale and cognitive level showed a good model fit (*X*^*2*^ = 33,01, *p* < 0.001; Nagelkerke *R*^2^ = 0.63). Indeed, 88,7% of the children were correctly classified by the model. However, only the PDP scale proved to be a significant predictor of diagnostic status with a *β* coefficient of 0.17 (*p* = 0.03), whereas the contribution of the cognitive level was not significant, with a *β* coefficient of − 0.53 (*p* = 0.06).

#### Correlation Between CBCL 1½-5 Scales and ADOS-2

A statistically significant positive correlation was found between the ADOS-2 total score and the CBCL 1½-5 Withdrawn scale (*r* = 0.57; *p* < 0.01), PDP scale (*r* = 0.45; *p* < 0.05) and ADHD scale (*r* = 0.42; *p* < 0.05), indicating that higher scores on these scales are associated with higher ADOS-2 total scores at 18 months.

#### ROC Analyses

**Fig. 2:**
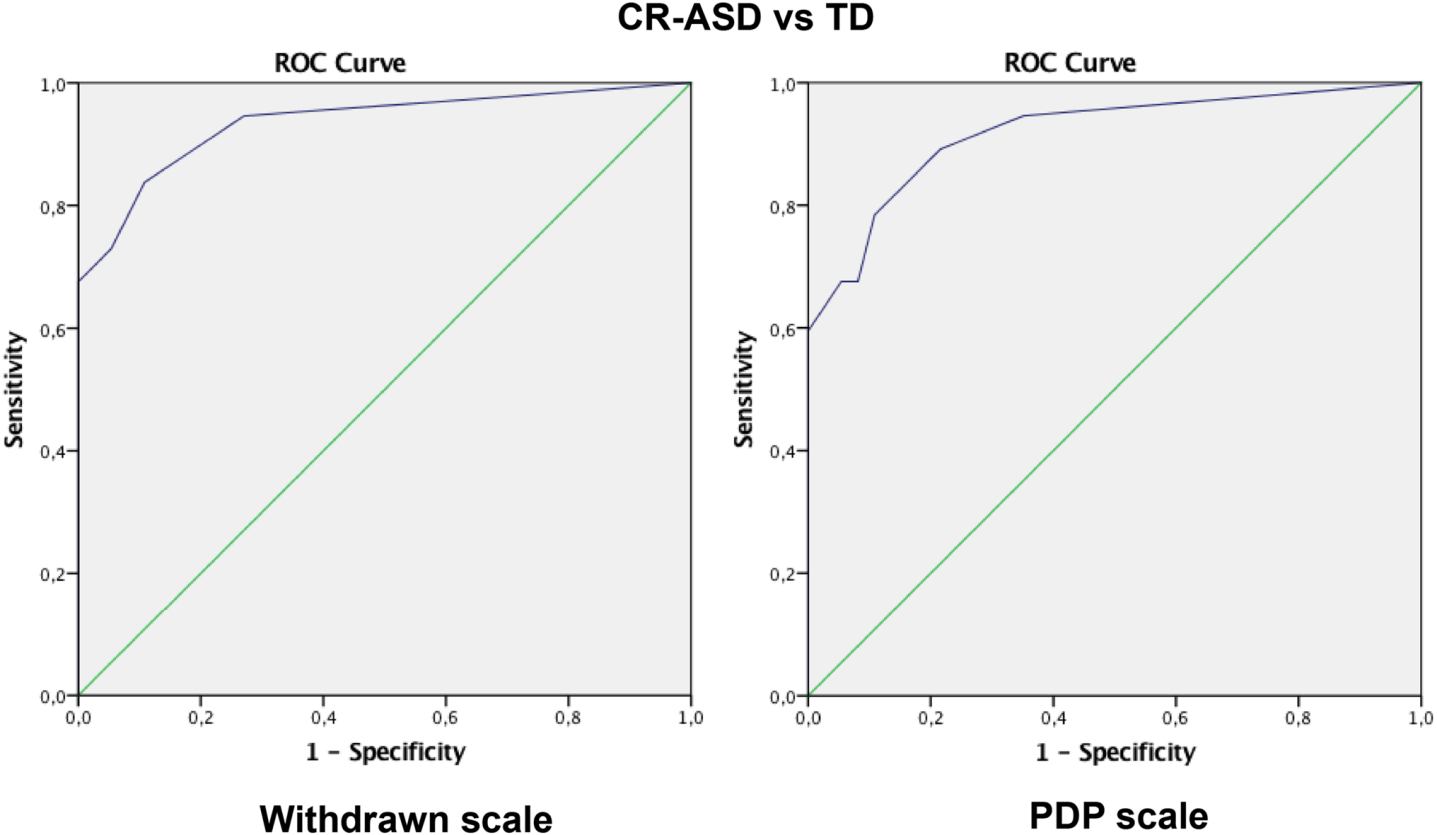
Receiver operating curve (ROC) for the withdrawn and PDP scales across clinically referred children for ASD (CR-ASD) and children with typical development (TD)

The area under the curve (AUC) was 0.94 (*p* < 0.001) for the Withdrawn scale and 0.92 (*p* < 0.001) for the PDP scale (*p* < 0.001), indicating that they are a valid tool for distinguishing between ASD children and TD children (see Fig. 2).

### Study 2: SIB-ASD vs. SIB-NonASD vs. TD

#### Comparison Between CBCL 1½-5 Profiles

After correcting *p*-values for multiple comparisons no significant difference emerged in CBCL 1½-5 profiles between the SIB-ASD, SIB-NonASD and TD groups using the Kruskall-Wallis test neither with the CBCL 1½-5 T scores nor with the standardized residual scores controlling for the effect of cognitive level (see the comparisons between *T* scores in Table 3; see the comparisons between standardized residual scores in Table 4).

**Table 3:** Comparison between siblings with a diagnosis of ASD (SIB-ASD), siblings without a diagnosis of ASD (SIB-NonASD) and children with typical development (TD) on the T scores of the CBCL 1½-5 scales: means, standard deviations and χ^2^ value of the Kruskall-Wallis analysis

	TD		SIB-ASD		SIB-NonASD		SIB-ASD vs. SIB-NonASD vs.TD	
	(*n* = 27)		(*n* = 10)		(*n* = 36)			
	*M*	(*SD*)	*M*	(*SD*)	*M*	(*SD*)	*χ*^*2*^	*p value*
Syndrome scales								
Emotionally reactive	51.17	(3.04)	50.6	(1.58)	51.19	(3.89)	4.50	0.105
Anxious depressed	50.71	(1.90)	51.7	(4.03)	52.03	(3.64)	0.35	0.838
Somatic complaints	51.66	(4.06)	52.3	(5.12)	51.47	(3.84)	0.29	0.864
Withdrawn	51.15	(2.66)	52.3	(5.19)	50.78	(2.49)	9.97	0.007
Sleep problems	52.92	(4.06)	51.8	(3.23)	53.69	(4.61)	2.26	0.323
Attention problems	52.02	(3.31)	53.2	(6.30)	51.53	(2.69)	0.24	0.885
Aggressive behaviors	50.75	(1.77)	50.1	(0.32)	52.33	(5.60)	2.39	0.303
Broadband scales								
Internalizing problems	41.19	(8.24)	43.6	(7.89)	38.97	(9.96)	7.33	0.026
Externalizing problems	42.69	(7.51)	43.3	(5.06)	44.36	(9.75)	0.01	0.996
Total problems	42.31	(7.04)	41.9	(6.62)	42.81	(9.61)	1.38	0.501
DSM-oriented scales								
DSM-affective	51.81	(3.06)	51.8	(2.3)	52.58	(3.78)	0.83	0.660
DSM-anxiety	50.85	(2.16)	51.5	(3.24)	52.25	(4.95)	0.21	0.900
DSM-PDP	51.46	(3.18)	51.9	(4.99)	51.00	(3.01)	5.75	0.056
DSM-ADH	51.93	(3.12)	51.7	(1.77)	52.44	(4.25)	0.43	0.808
DSM-ODP	50.61	(1.76)	50.1	(0.32)	51.53	(3.52)	2.64	0.268

The level of significance was corrected for multiple comparisons in agreement with Bonferroni’s procedure (*p < 0.003)

**Table 4:** Comparison between siblings with a diagnosis of ASD (SIB-ASD), siblings without a diagnosis of ASD (SIB-NonASD) and children with typical development (TD) on the standardized residual scores of the CBCL 1½-5 scales (Cognitive Level as Predictor): means, standard deviations and χ^2^ value of the Kruskall-Wallis analysis

	TD		SIB-ASD		SIB-NonASD		SIB-ASD vs. SIB-NonASD vs.TD	
	(*n* = 27)		(*n* = 10)		(*n* = 36)			
	*M*	*(SD)*	*M*	*(SD)*	*M*	*(SD)*	*χ*^*2*^	*p value*
Syndrome scales								
Emotionally reactive	− 0.03	(0.73)	− 0.02	(0.54)	0.03	(1.25)	7.46	0.024
Anxious depressed	− 0.19	(0.75)	− 0.00	(1.20)	0.14	(1.09)	3.34	0.189
Somatic complaints	0.12	(1.09)	− 0.02	(1.16)	− 0.08	(0.88)	9.82	0.007
Withdrawn	0.06	(0.89)	0.34	(1.69)	− 0.14	(0.81)	4.25	0.120
Sleep problems	− 0.09	(0.93)	− 0.20	(0.79)	0.12	(1.09)	0.02	0.991
Attention problems	− 0.07	(0.82)	0.49	(1.80)	− 0.09	(0.78)	2.66	0.265
Aggressive behaviors	− 0.18	(0.50)	− 0.19	(0.12)	0.19	(1.33)	4.59	0.101
Broadband scales								
Internalizing problems	0.26	(0.82)	0.21	(0.87)	− 0.25	(1.10)	7.02	0.030
Externalizing problems	− 0.05	(0.90)	− 0.16	(0.65)	0.09	(1.14)	0.13	0.939
Total problems	0.06	(0.80)	− 0.02	(0.78)	− 0.04	(1.18)	0.73	0.696
DSM-oriented scales								
DSM-affective	− 0.16	(0.92)	− 0.03	(0.66)	0.13	(1.12)	3.23	0.199
DSM-anxiety	− 0.23	(0.36)	− 0.03	(0.85)	0.18	(1.30)	4.67	0.097
DSM-PDP	0.09	(0.87)	0.26	(1.49)	− 0.14	(0.92)	6.86	0.032
DSM-ADH	− 0.12	(0.67)	− 0.05	(0.52)	0.10	(1.27)	2.49	0.289
DSM-ODP	− 0.18	(0.64)	− 0.18	(0.16)	0.18	(1.28)	3.51	0.173

The level of significance was corrected for multiple comparisons in agreement with Bonferroni’s procedure (*p < 0.003)

#### Correlation Between CBCL 1½-5 Scales and ADOS-2

Spearman’s correlation did not find any statistically significant coefficients between parent ratings on the CBCL 1½-5 and clinician observations at the ADOS-2.

## Discussion

This study aimed to explore whether the CBCL 1½-5 could provide useful information for identifying children at risk for ASD as early as 18 months. Our results (Study 1) show that the CBCL 1½-5 Withdrawn and PDP scales can differentiate children with ASD from children with TD at this early age. Furthermore, group membership (ASD vs. NonASD) was predicted by the Withdrawn and PDP scale *T* scores, but not by the level of cognitive ability. We also found that higher scores on these scales correlated positively with the clinician’s assessment of autism with the ADOS-2 semi-structured observation. These results confirm findings from previous studies on older children with ASD. Indeed, both the DSM-PDP scale and the Withdrawn Syndrome scale have shown an ability to differentiate children with ASD from children with TD at 24 months (Rescorla, Winder-Patel et al. 2019b), between 18 and 36 months (Narzisi et al. 2013), and between 24 and 60 months (Muratori et al. 2011). These results are not surprising as the two scales have five overlapping items. However, the DSM -PDP scale includes more specific ASD-like behaviors than the Withdrawn scale (i.e. 63. Repeatedly rocks head or body, 80. Strange behavior) and has been reported to have higher sensitivity compared to the Withdrawn scale (Levy et al. 2019).

Although screening at 18 months vs. 24 months or later ages has the potential to significantly accelerate the diagnostic process, there is a risk that some children with milder traits may be screened negative at this young age. Indeed, in their sample of 120 children with ASD, Zwaigenbaum et al. 2016 found that only 16% were diagnosed correctly at 18 months, 46% received their diagnosis at 24 months, and another 38% at 36 months, with children with more advanced language and adaptive skills and milder ASD symptoms being diagnosed later. If accuracy is a priority for Level 2 screening, this is not the case with Level 1 screening, which seeks to maximize sensitivity in order to avoid missing potential cases (few false negatives), accepting that some children will be false positives (they may have other behavioral/emotional problems that need attention).

Nevertheless, in our study, the ability of the CBCL 1½-5 to differentiate between children who are developing ASD and their peers with TD appeared specific to the clinically referred group. In the other at-risk group (Study 2), composed of children at familial risk for developing ASD due to an older affected brother/sister, the CBCL 1½-5 had difficulty in differentiating correctly between siblings who were developing ASD and the control group of children with TD. When the effect of cognitive level was removed, and the groups were matched on cognitive level, by using the standardized residuals of the *T* scores with cognitive level as predictor, no significant differences appeared between groups. The SIB-ASD CBCL 1½-5T scores were below clinical cut-offs and quite similar to the control group of children with TD as well as to the SIB-NonASD group (see the *T* scores on the CBCL 1½-5 scales of the three groups SIB-ASD, SIB-NonASD, and TD in Table 3).

Our results on the use of the CBCL 1½-5 in siblings differ from findings in a previous study by Rescorla, Winder-Patel et al. (2019b), who in a similar small group of 13 SIB-ASD children found higher scores on the Withdrawn and DSM-PDP scales in siblings diagnosed with ASD compared both to low risk children and to siblings without a diagnosis. As their study was conducted on older children (24 months of age) than our toddlers, it is possible that by the time the children had reached their second birthday atypical behaviors may have become more evident for parents who filled in the CBCL 1½-5. Furthermore, Rescorla does not quantify the ADOS-2 scores of the children in her sample, so we were not able to compare our data with hers regarding the severity of autistic profiles.

Conversely, the characteristics of our SIB-ASD sample do not appear particularly different from other descriptions of siblings with ASD of the same age. In Chawarska’s study on predictors of later outcomes in younger siblings of children with ASD, the mean ADOS-2 severity score index of 69 SIB-ASD children who were correctly identified at 18 months was 6 and increased to 7 at 36 months (Chawarska et al. 2014). Our severity score index of 6.7 indicates that the symptomatology of our SIB-ASD sample was not particularly low and was recognized quite clearly by clinicians at the ADOS-2 semi-structured observation. Cognitive and adaptive functioning were significantly lower in our SIB-ASD group than in the SIB-NonASD group, although on average they did not reveal a clinical delay (mean scores were above 70 on all subscales). These profiles are similar to those presented in other studies on siblings’ developmental trajectories which show a slower developmental rate in SIB-ASD children (Landa and Garrett-Mayer 2006; Sacrey et al. 2019).

Our results regarding the difficulties of the CBCL 1½-5 to clearly identify autistic symptoms in the siblings were partially unexpected. Firstly, because this instrument proved useful in clinically referred children of the same age (Study 1) and secondly, because parents of autistic children have generally been shown to be sensitive to their younger children’s development (Herlihy et al. 2015; Richards et al. 2016; Sacrey et al. 2015). The different discriminative capacity of the CBCL 1½-5 in our two studies might be explained by differences in the ascertainment method of the two groups. Indeed, children who are recruited in prospective longitudinal studies are more likely to display fewer and less severe symptoms than those recruited on the basis of clinical referral or with a provisional diagnosis (Sacrey et al. 2017). Thus, it is possible that with individuals of this kind, screening instruments whose properties include greater variance in the distribution of features are more informative (Pasco et al. 2019).

Furthermore, although it has been shown that parents of children subsequently diagnosed with ASD are more likely to report concerns about their child’s development than parents of children with TD and children with other developmental difficulties, their concerns tend to be more about broad behavioral issues rather than about social communication and interaction (Pasco et al. 2019). If, on the one hand, parents who have older children with ASD are inevitably better informed about the emerging signs of autism than most parents of young children, it is possible that when comparing their younger offspring with the older child with autism rather than to “typical development” they may tend to under-report autistic-like behavioral symptoms, especially when they differ from the older sibling’s behavioral profile. Indeed, in our sibling group the SIB-ASD group showed unexpectedly low scores on the CBCL 1½-5 and SIB-NonASD children scored even lower. Inconsistencies between parent reports of autistic traits and observations by other informants such as teachers or clinicians are quite common and should not be considered as contradictory but as complementary, as each informant provides unique information based on their specific experiences or situational specificity (Möricke et al. 2016). Indeed, Nilsson Jobs et al. (2019), who tested the efficacy of the CBCL 1½-5 in siblings at heightened risk of developing ASD, found that teachers’ reports of autistic symptoms increased the likelihood of correctly differentiating between siblings with and without ASD. In the light of these observations it is possible that in sibling populations clinicians may benefit by asking multiple informants to fill in the CBCL 1½-5.

When interpreting the results of the present study important limitations should be taken into account. The main limitation of this work is the small group size of the SIB-ASD group. However, this is a fairly common limitation in sibling studies and our group size is similar to the ones in the two previous studies on the use of the CBCL 1½-5 in sibling populations (n.13 Rescorla, Winder-Patel et al. 2019b; n.10 Nilsson Jobs et al. 2019). Despite the limited sample size, we believe that the inclusion of this group of children is important. Indeed, it provides complementary information, not limited to clinically referred toddlers whose parents are already aware of the reasons for concern, on the use of the CBCL 1½-5 in toddlers who are at risk for autism. Nevertheless, generalizability of the findings from Study 2 should be addressed with caution as non-significant results could be the result of the small sample size and less powerful statistics. The wide variability of ASD traits within children at familial risk and the young age of the children could also have contributed to this result. Indeed, in their study on high-risk siblings Rescorla, Winder-Patel et al. (2019b) suggest that a lower DSM-PDP cut-off point might be preferable when screening for ASD at a young age, when symptoms may be more subtle or less severe. Another possible limitation regarding our non-significant findings for study 2, may be related to the fact that the computation of the *T* score of the PDP scale also included an item recently excluded (item 3, *afraid to try new things*) as it did not meet the threshold for inclusion in the DSM-5 version of the scale. Indeed in our sample of siblings who received a clinical diagnosis of ASD, only 20% of parents reported some kind of problem on item 3. However this error was evenly distributed across children (e.g. also in study 1, where the CBCL correctly identified children with autism, only 45% of children obtained a higher score than 0 on item 3). Another limitation is the fact that no follow-up data is available from the TD group in order to ascertain developmental outcome. However, at the moment of recruitment there were no clinical concerns regarding these children’s development. Furthermore, a measure of cognitive ability was not available for all the children in the TD group, so we selected a reduced number of children with TD (n.27) for Study 2 and we were only able to control for the effect of cognitive level in a sub-group of children in Study 1. In the future, it would be useful to include also a group of children with developmental delay (DD), in order to better evaluate the effects of cognitive level on ASD screening. In previous studies a higher rate of false positives has been reported in DD groups (Rescorla et al. 2015; Havdahl et al. 2016). However, Levy et al. 2019 found that the CBCL 1½-5 screening capacity was higher among DD children who did not share ASD features than among DD children who had ASD features (44% of their DD sample), indicating the importance of considering phenotypical differences among DD children.

In conclusion, our findings suggest that when parents raise concerns for ASD by presenting high scores on the Withdrawn and PDP scales, an evaluation for ASD is highly recommended as there is a strong likelihood that the child may have the disorder. We believe our preliminary study lays the foundation for a future population study, which could better verify the discriminative capacity of this instrument at the 18-month well-child visits.

However, when looking at families who already have a child with ASD, we found low agreement between parent ratings on the CBCL 1½-5 and the diagnostic assessment performed by the clinician. Thus, we strongly recommend that younger siblings of children with ASD be followed in longitudinal surveillance programs. Moreover, multiple sources of information should be collected in order to gain a more exhaustive picture of the child’s communication and social development.

## Appendix

See Table 5

**Table 5:** List of the items composing the DSM-pervasive developmental problems scale and the withdrawn syndrome scale; overlapping items are evidenced in bold; item 3, which has been removed from the DSM-5 version of the CBCL 1½-5 is presented between brackets

	DSM-PDP scale		Withdrawn Syndrome scale
		2	Acts too young for age
3	[Afraid to try new things]		
**4**	**Avoids looking others in the eye**	**4**	**Avoids looking others in the eye**
7	Can’t stand having things out of place		
21	Disturbed by any change in routine		
**23**	**Doesn’t answer when people talk to him/her**	**23**	**Doesn’t answer when people talk to him/her**
25	Doesn’t get along with other children		
		62	Refuses to play active games
63	Repeatedly rocks head or body		
**67**	**Seems unresponsive to affection**	**67**	**Seems unresponsive to affection**
**70**	**Shows little affection toward people**	**70**	**Shows little affection toward people**
		71	Shows little interest in things around him/her
76	Speech problem		
80	Strange behavior		
92	Upset by new people or situations		
**98**	**Withdrawn, doesn’t get involved with others**	**98**	**Withdrawn, doesn’t get involved with others**

.
